# The Effect of Maternal Behavior around Calving on Reproduction and Wellbeing of Zebu Type Cows and Calves

**DOI:** 10.3390/ani11113164

**Published:** 2021-11-05

**Authors:** Agustín Orihuela, Carlos S. Galina

**Affiliations:** 1 Facultad de Ciencias Agropecuarias, Universidad Autónoma del Estado de Morelos, Cuernavaca 62209, Mexico; 2 Departamento de Reproducción, Facultad de Medicina Veterinaria y Zootecnia, Universidad Nacional Autónoma de México, Ciudad Universitaria, Ciudad de México 04510, Mexico; cgalina@unam.mx

**Keywords:** welfare, cow-calf bond, peripartum, *Bos indicus*, tropics

## Abstract

**Simple Summary:**

After a brief introduction establishing the importance of parturition and the establishment of the mother-young bond, this review summarizes knowledge on the maternal behavior of Zebu type cows, including maternal and young behavioral patterns, the protective behavior of the mother and how maternal behavior might be influenced by experience and weather conditions. In addition, the effect of some nutrition, heat stress, calf presence around parturition and its effects on cattle reproductive performance, welfare issues on reproductive behavior and calf performance are reviewed. Finally, some conclusions and practical implications are established.

**Abstract:**

The behaviors associated with domestic cattle such as maternal care are quite similar to those behaviors observed in wild ungulates. These behaviors allow the cow to bond with her calf, protect and provide it with nourishment and ultimately reduce the bond at weaning. Although maternal behavior is an important factor influencing the survival and early development of the newborn calf, Zebu type cows around calving have not been studied extensively. Herein, we consider the four main aspects of maternal behavior in cattle and particularly *Bos indicus* cows and calves. Firstly, we provide a brief description of the behavior of cows around parturition and the behavior of the first stages of the calves’ lives. In the second part, the protective behavior of the mother is analyzed. Subsequently, examples of animal welfare implications followed by an analysis of some factors that affect calf survival, including mother experience and weather conditions, are discussed, and in the last part, reproduction along with some peculiarities of reproductive behavior, and the wellbeing of mother and calves are examined. We concluded that knowledge of maternal behavior of Zebu type cows around calving and interactions with calves might contribute to an enhanced reproductive efficiency of the mother and the welfare of the calf.

## 1. Introduction

Once a Zebu cow becomes pregnant, she carries, in most of the cases, a single calf for about 283 days. Male calves are carried for 4 to 5 days longer, resulting in a higher birth weight. It has been estimated that each day longer in gestation results in a 1.4 kg increase in the birth weight [1].

Calves stand and walk shortly after birth, and cows nurse their offspring for about six to eight months, with other members of the herd playing a critical role in the protection of the calves. However, the attention for the calves is provided primarily by their mothers [2]. Parental care entails how much energy is necessary to invest in a neonate at the cost of the reserves of the cow’s resources for her calf [3], besides for her own reproductive performance. The cow after parturition is the most important social individual for the calf, providing protection against predators, food, warmth, shelter and immunological defense [4,5]; thus, important information concerning the physical and social environment is essential for the calf´s survival. At the same time, the presence and nursing behavior of the calf coupled with other environmental conditions including the body reserves of the mother, affect the interval from parturition to the resumption of the ovarian activity.

## 2. Maternal Behavior

### 2.1. Behavioral Patterns

When cattle are kept free under extensive conditions, it has been observed that there is a certain organization among the members of the herd according to their physiological state. Pregnant cows tend to form more cohesive groups that walk greater distances, away from calves and other cows [6].

Pregnant cows usually isolate themselves from the herd when near calving [7]. Arching of the back and an elevated tail occur for 1 to 3 h before the chorioallantoic membranes rupture. During this period, many cows licked or kicked their flanks. Some of the signs of imminent parturition are relaxation of the sacrosciatic ligament, slackening of the tissue of the perineum and vulva, distention of the udder and teats and mucous discharge from the vulva.

Labor onset occurs about 2.5 h before calving. During the birthing process, it is common for pregnant cows to lie down and stand frequently [8]. The identification of this type of behavior may have practical relevance, since the risk of a calf dying at birth is higher when the cow calves in a standing position (16%) compared to lying down (4.2%) [9]. This feature is particularly important in cattle calving at pasture. When membranes rupture, the cow often licks the fluid and tends to stay near the spot, now attractive to the cow, where the fluid fell. The discharge is followed by the appearance of the calf’s feet. The calf is completely delivered during a period of 45 to 290 min, and time is often used as a measure of labor difficulty. Most calves are delivered in an extended posture, dorsal position and anterior presentation. Zebu calves weigh an average of about 25 kg, with variations between breeds.

Placenta expulsion can take place immediately after parturition or up to five hours later, with a tendency to involve more time in male calves. The placenta weights between 2 and 4.5 kg with 41 to 112 placentomes per placenta [1,10]. The placenta is eaten by about 82% of cows [11].

**Figure 1:**
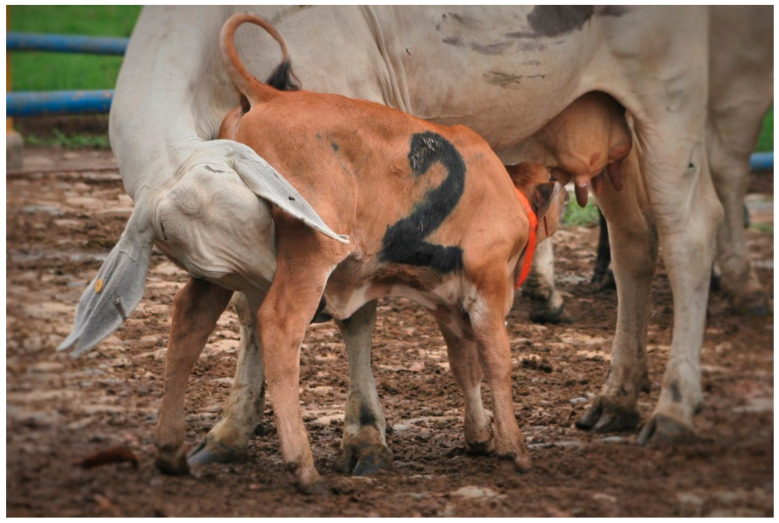
Postpartum cows allow their calves to suckle, which they identify through opposing signals favored by the calf’s normal suckling position.

The newborn calf shakes its head, snuffles and sneezes. This behavior may begin during parturition as soon as the calf´s shoulders are free of the mother´s vulva. Some calves will remain motionless for up to 30 min after birth, but within an hour most calves can stand. It may take 30 min to an hour before the teats are located, and the cow´s conformation may not provide the higher recess that the calf appears to seek. Most calves suckle within 3 h (Figure 1).

Some previous studies described the occurrence of allosuckling in cattle, ranging from 3.0 [12] to 19.02% of the total suckling events [13], but it seems to be rare in Zebu cattle [14]. In general, ungulates have been classified as hiders and followers [15]. Bovines, including *Bos indicus* cows, are species where the young remain at or near the birth site for 3 to 7 days after parturition, whilst the mother may be away for long periods of time [16]. The young of this kind of animal can afford to have a low degree of social responsiveness, since the mothers actively seek them out at feeding times [17]. After this hiding period, Orihuela et al. [18] demonstrated that newborn Zebu calves frequently form groups with one or more cows. These researchers observed 142 groups, where most of them were formed by 1 to 3 cows and 2 to 32 calves between 4 and 32 days old. This kind of communal rearing may be a strategy developed to allow different cows to move for better access to feeding while their calves remain in larger groups and are cared by other adult cows [19]. Additionally, this feature indirectly improves maternal energy budgets [20]. This may also be an anti-predatory behavior [21,22], facilitating calves’ socialization [23]. These communal groups of several calves with some cows might be favored by the fact that cows group according to their physiological state, affecting the way herd members interact with each other [6].

Similarly, Reinhardt and Reinhardt [24] studied the cohesive relationships in a *B. indicus* cattle herd over a 3–5-year period and concluded that the social structure is based on matriarchal families that are interconnected by means of friendship relationships between non-kin partners. In addition, Enriquez et al. [25] demonstrated that 33% of 25-day postpartum Brahman cows, during a maternal protective test, reacted when their calves or calves from other cows individually walked freely for 30 s in front of the continuous pen where the cows were held.

In cattle, the suckling bout lasts for some time and includes additional communication. Before milk ejection, there is about one minute of suckling and butting; afterwards, there is a period of several minutes of continued nursing from different teats and some butting, that might partly be a way for the calf to stimulate further milk production [26], and thereby, communicate nutritive needs to the dam [17]. Particularly in Zebu type cattle, it has been demonstrated that the tactile stimulation provided by the calf improves milk ejection and milk yield [27]. Some early studies showed that in Zebu breeds of cattle, milk ejection cannot be activated in the absence of a suckling calf [27] and, if the calf dies, blowing in the cow´s vagina [28] or presenting a dummy of a calf´s head [29] is necessary to obtain milk. For this reason, many herdsmen of Zebu cattle allow the calf to suckle first, to induce the milk let-down reflex [30].

### 2.2. Protective Behavior

In general, recently calved cows are considered dangerous and aggressive animals, especially *B. indicus* breeds [31,32], where the maternal aggression of mothers protecting their newborns is considered one of the main causes of accidents with cattle [33,34,35]. It has been proposed that this may be a consequence of an increase in their maternal protective behavior or a modification of their temperament triggering nervous responses, as well as the maintenance of an alertness attitude [4]. Orihuela et al. [36] found that the temperament of the cows did not affect their reaction to people handling their calves during the first days postpartum. This finding suggests that temperament measures cannot be used to predict the aggressiveness of the cows; however, there seems to be a genetic reason that explains the difference in aggressiveness at parturition within several beef cattle breeds [37].

Aggressive cows can be a high risk to handlers. Sometimes it is necessary to approach cows during parturition to intervene in some dystocia problems, and in general, newborn calves require care soon after delivery to treat navels and apply some identification procedure, which is essential to ensure good health and reduce the mortality rate of the newborns. It is during these last approaches that some cows, and in general those that give birth for the first time, might become nervous and sometimes show aggressive behavior towards the handlers. This reaction could be interpreted as a natural response to a potential predator that threatens her young. Aggressive cows can be a real problem. Costa [38] evaluated 4385 Nelore cows, finding this behavior in 13.2% of the cases, where 11.4% threatened and 1.8% attacked their handlers, which can be potentially risky in poor installations when working under extensive conditions. Negative interactions between animals and handlers lead to situations of stress and fear, increasing the risk of aggressions. Thus, de Oliveira Costa et al. [39] propose training handlers to improve the human-animal relationships, the use of adequate facilities and the slaughter of dangerous animals to solve this problem.

The possible presence of predators at the parturition place also affects the cow–calf relationship. Toledo [40] has given special attention to species such as the black vulture (*Coragyps atratus*) because in some countries the presence of these animals increases around the calving sites, interfering with the normal establishment of the cow–calf bond, in addition to direct attacks on the calves. The main effect that has been caused is a reduction in the time that the mother spends in contact with her offspring. This ends up negatively affecting the difficulties that newborns might have to stand up and suckle [40]. 

In cattle production systems, handling the calf at an early age is necessary for routine management procedures [41]. It is possible that cattle perceive humans as potential predators, and thus, cows may exacerbate their reactions [42].

### 2.3. Mother Experience and Weather Conditions

Some of the factors that affect the position of the cow at calving are the lack of calving experience, and the presence of potential predators around the calving site [7,40]. Episodes of aggressive behavior toward the newborn calf are also important. In accordance with Schmidek et al. [43], these events are more frequent in primiparous than multiparous cows (55.7 and 22.0%, respectively), resulting in a higher percentage of calves that do not achieve their first suckling within the first 3 h postpartum (15.7 and 5.7%, respectively) and in greater latencies for a first successful suckling (102.6 and 76.0 min, respectively). The risk of mortality in the neonate also increases with the delay of a first postpartum suckling for more than 3 h [43]. Weather conditions [44], calf weight at birth, udder shape and teat size [43] are other factors that could also result in a failure or delay of the first postpartum suckling. Up to a third of calves may not suckle within 6 h of birth. This is particularly apt to be the case when the cow has a pendulous udder or thicker teats, such as the Indobrasil breed [45,46]. 

In research carried out with 1527 calves born between 1992 and 2004, it was found that 279 (18.3%) failed to achieve their first suckling and required assistance to be able to suckle after 6 h of being born. Hanging udders and large teats, as well as calves weighing less than 25 kg at birth, were the two factors identified as the main causes of failure to achieve the first postpartum suckling [47]. Therefore, in practice, the conformation of the udder of the cows and the weight at birth of the calves become possible indicators that these animals will require help for the survival of the calf. 

As mentioned above, Zebu calves congregate in newborn groups that also integrate at least one adult cow, preferably higher parity cows [18]. Older cows have more experience in calf care, which could influence some alloparent behavior, along with other physiological differences such as increased milk production and a higher number of antibodies in their colostrum than younger mothers [48].

In general, younger parents seem to have a higher residual fitness cost for reproduction and a poorer probability of raising young successfully and tend to show less parental care [49]. In a recent study, Enriquez et al. [25] found that during a maternal protective test, cows with slight follicular development reacted to a greater number of calves displaying a higher intensity of reaction. In addition, these cows did not display estrous behavior, suggesting a relationship between protective maternal behavior and some reproductive variables in Zebu-type cows (*B. indicus*). This seems to be in accord with the theory of terminal investment, where animals with a favorable potential to reproduce, would do so [49]. 

## 3. Reproductive Performance

Rust [50] suggested that extensive production systems will be under considerable pressure because of land availability and predicted climatic changes. Thus, there is a need to intensify production systems in the cattle industry [51]. This scenario has moved producers and scientists alike to find methods to change the traditional strategy, to leave the cows at pasture and collect the offspring at defined periods. Consequently, the economic and reproductive efficiency of the cows are influenced by the survival of their calves, as their livelihood and performance are the main source of income for beef cattle producers [52]. Knowledge about maternal and filial behavior is important to identify those situations that could increase the risk of weak, abandoned or stillborn newborns, since it offers the opportunity to reduce these types of problems, thereby reducing economic losses [47,53]. 

Orihuela and Galina [54] concluded that any method of calf separation should consider factors such as the time when the event is performed, the nutritional status of the cow and the effect of stress on the dam and the offspring. Breaking the cow–calf bond in the different methods utilized need to be reviewed, not only from the reproductive viewpoint but also from the welfare of the animals [55].

### 3.1. Nutrition and Onset of Ovarian Activity

Animals raised under pasture conditions depend on adequate fodder to gain weight after calving and have enough milk for their young [52]. This is not an easy feat considering that animals tend to calve in the spring, usually prior to the rainy season, where the fodder available in the last trimester of pregnancy could affect the onset of ovarian activity [56,57]. In effect, in a comparative study between two locations in the tropics of México and Costa Rica where the onset of the rainy season could vary, it was found that the pattern of body loss in the last trimester of gestation was directly related to the onset of the rainy season [58]. In fact, in a further experiment, when two groups of cows with different postpartum periods were subjected to a synchronization program, monitoring body fat change via ultrasound, it was found that the driving force for a prompt restoration of ovarian activity and pregnancy were the changes in body reserves rather than the time postpartum [56,57]. These examples illustrate the need for farmers and technicians alike to join resources to avoid body loss during the last trimester of gestation catering for the wellbeing of the dam and later for their offspring. This principle is hardly dependent on having an adequate use of the pasture available to avoid an unfair competition between animals of different weights and calving. First calf heifers are usually at a higher risk [59,60]. Burns et al. [61], in an extensive review about factors affecting the reproductive efficiency of beef cows in Northern Australia, underline the importance of nutrition as a key player for a prompt restoration of ovarian activity. A failure to meet nutritional requirements would cause, in those cows, calving intervals longer than 15 months.

There are alternatives to improve pasture availability and animal welfare in the tropics. For example, the use of native trees and shrubs [62] facilitating the shading area and soil restoration; therefore, pastures will be available and not dried out to direct solar radiation for longer periods of time [63].

Amendola et al. [64] performed an interesting experiment comparing a group of heifers grazing in a silvopastoral scheme as opposed to a monoculture system during two distinct seasons of the year in the dry tropics of Mexico, concluding that the hierarchy in the silvopastoral system was more constant and stable between seasons compared to the monoculture system. The same group [65] compared the effect of improved pastures and shaded areas in the hottest months of the year, as opposed to the traditional system of monoculture, suggesting that forage availability and access to shade allow cattle to have longer periods of rest, permitting a better rumination and general performance. These experiments were carried out in heifers; therefore, how such factors affect mother and offspring relationships remains to be elucidated.

This is particularly important in dams recently calving. The stress produced by parturition, suckling of the newborn, plus the need to search for a calving site and protect their calves from predators, are monumental tasks for the dam, still needing to search for adequate pastures to sustain their body metabolism.

A different story is the situation of cattle known as dual purpose, which is estimated to be present in 70% of the livestock living in the tropical and sub-tropical regions of the world [66]. This system started with crossbreeding Holstein or Brown Swiss with Zebu breeds, mainly Brahman, Guzerat and Gyr plus other local undefined Zebu type of cattle. This mélange of breeds has caused difficulties in characterizing their reproductive capacity, as the feeding regimens consisted of local or improved grasslands and various combinations of feeding supplementation [67].

This system is popular due to several factors related to their adaptation to the harsh environmental conditions in the tropics. There is less capital investment and technical support [66]; thus, it is not surprising that their level of technology adoption is so varied and heavily dependent on their location [68]. All these factors make it quite complicated to draw conclusions to the wellbeing of the calf and dam either by judging their nutritional requirements or reproductive efficiency.

### 3.2. Heat Stress

Breaking the mother–offspring bond can affect the welfare of both. Although, there is abundant information on the different methods to separate the calves from their dams (for an early review see [69]). In comparison, few control studies are available on the stress and their ultimate welfare. It has been shown that rising levels of cortisol have an important effect on the reproductive performance of the dams [70]. Additionally, raising animals under extreme heat environments affects their reproductive performance, influencing the wellbeing of livestock in general. Bernabucci et al. [71] have postulated that heat stress is caused when the body temperature of an animal is beyond its normal range, creating a difficulty in heat dissipation and finally reducing physiological as well as behavioral responses. Bearing in mind that beef cattle in the tropics are usually exposed to a high number of hours, and in many enterprises, without shelters for the animals, Diaz et al. [72] studied the effect of higher temperature indexes (THI) and found a negative effect on the resumption of ovarian activity, especially if these higher THI occurred during the last trimester of pregnancy. This study also underlined the importance of some animals being able to restore ovarian activity despite a hostile environment. How do high temperatures affect the performance of cattle during calf separation? A question in need of answers.

What is certainly known, is that *B. indicus* cattle are more resistant to high temperatures than *B. taurus*. Beatty et al. [73] suggested that *B. taurus* breeds have less resistance to heat stress, a subsequent reduced feed intake and raised plasma non-esterified fatty acids compared to *B. indicus*. The ability to tolerate heat in *B. indicus* is manifested in their reproductive ability to raise follicles capable of fertilization [74]. Even more, Blackshaw and Blakshaw [75], in a detailed review, point out that the advantages of *B. indicus* cattle over *B. taurus* are related to physiological events, such as the ability to disperse heat due to the coat color of their skin, food and water intake and sweating intensity. Most of these research communications are related to a comparison between *B. taurus* and *B. indicus*. It would be commendable to compare different breeds of *B. indicus* to find out if effectively all the breeds are similar in their behavior, particularly if the management system relies on allowing the calf to suckle at will.

### 3.3. Presence of the Calf

**Figure 2:**
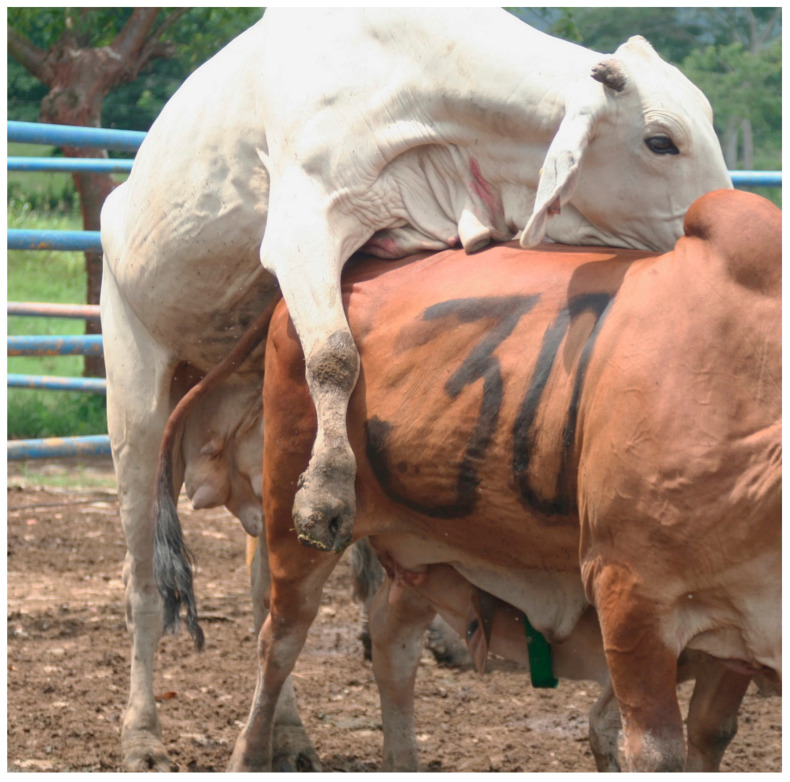
Two lactating cows display sexual behavior. The cow in the bottom shows sexual receptivity even while nursing. Reproductive activity in Zebu cows can be induced after a period of separation between the mother and her calf, inhibiting the negative effect of suckling and the presence of the offspring on the resumption of postpartum ovarian activity.

There seems to be a common agreement that suckling and the presence of the calf impaired the adequate growth of a follicle capable of producing a viable zygote [76]. This shortcoming is even more evident when the animals are under stress [77]. Nonetheless, calf separation remains the single most important tool to restore ovarian activity after calving [78] (Figure 2).

Whatever the method used to break the bond between the dam and the offspring, it can be strengthened with the use of hormonal therapies to promote follicular growth [79]. Mondragon et al. [80], in a detailed study of the follicular dynamics comparing animals under a regimen of restricted suckling (RS) with the continuous presence of the calf, observed that cows in the RS treatment had a larger follicle than the control cows. The authors concluded that interrupting suckling will favor the development of healthier follicles. Furthermore, Álvarez-Rodriguez et al. [81] found that the management practices limiting suckling must also avoid a close cow–calf association to reduce long postpartum intervals to first ovulation.

A different issue in need of research relates to the diverse systems utilized in dual purpose cattle, starting from having the calf present, allowing the milk let down from the cow, to allowing the calf to suckle one teat or other combinations. In recent years, the introduction of more elaborate procedures, such as extracting the milk without the presence of the calf either by hand or machine are more common. No matter what system is used, it is not surprising to find calving intervals wider than 14 months in the literature [82,83]. More research is obviously in demand for human interventions [84]. One of these procedures is related to the beneficial effect of calf separation. Recent evidence suggests that neither calf separation nor hormonal therapies are strictly necessary to restore ovarian activity, since techniques such as restricted suckling have proven to be effective [85].

### 3.4. Sexual Behavior

The sexual behavior of Zebu cows offers certain specific characteristics, which must be taken into account when conducting heat detection programs, since on many occasions the manifestation of external signs of estrus can be affected by many factors [86,87]. Some of these factors are the complex social order and dominance characteristics in cows. For example, not all heifers in a herd mount cows in estrus [88]. Orihuela et al. [89] found that 60% of the mounts among females were performed by a heavier and larger cow than the one being mounted. Similarly, when evaluating a herd composed of Charolais and Brahman cows, it was observed that most of the mounts were carried out by Charolais cows on Brahman cows, which were comparatively larger and heavier than the latter, while Brahman cows rarely rode Charolais cows [90]. On the other hand, the larger, dominant cows inhibit the riding activity of the smaller, subordinate ones, sometimes even of young or inexperienced males [91]. However, a correlation between the dominance rank of the females in a herd and the number of mounts given or received, has not been demonstrated [92]. Other factors that affect the manifestation of estrus are breed, age, weight, presence of horns and the number of cows in the sexually active group [86,93]. Sexual receptivity, or allowing oneself to ride, remains the most unequivocal sign of estrus, and homosexual interactions are very common in Zebu cattle, coupled with the fact that mounting between Zebu females occurs almost exclusively when both animals are in estrus [94].

When the estrus of Zebu cows is synchronized artificially, either through the use of drugs or by separating the calf from her mother, or both, a high percentage of animals participate in sexually active groups, leading to a greater number of animals in estrus, with longer and more intense estruses, due to social facilitation and sexual stimulation. Orihuela et al. [95] found that by staying together, about 80% of the unsynchronized cattle showed signs of heat similar to those of their progestin-treated herd mates. Similar studies have programmed the induction of estrus in Zebu cows so that they display behavioral signs of estrus, one after another, every third day. In response, the cows grouped their riding behavior on the days for which they were scheduled [96]. Natural heat synchronization appears to be common in Zebu cattle against artificially synchronized herd mates [97]. Medrano et al. [96] found that 85% of the mounts detected after an estrous synchronization program are received and given by cows in estrus, although, due to imitation behavior, some of them exhibit active mounts in the absence of follicles capable of ovulating, affecting the final results of artificial insemination. The type of heat could be affecting the duration of estrus. However, researchers generally agree that the duration of estrus in *B. indicus* cattle is shorter than in *B. taurus* cattle [98,99].

Handling can also affect when estrus is displayed. Vaca et al. [100] kept Zebu cattle reared under extensive conditions in a pen to facilitate the detection of estrus and found that 50% of the animals did not show estrous signs until they were released to the pasture again. Likewise, during strong tropical storms, the Zebu cow can suspend the manifestation of sexual behavior.

Environmental temperature is also a factor, and in general, *B. indicus* cattle show sexual behavior more frequently during the hot summer months [101], compared to European cattle that manifest more in cold climates [102]. However, although Zebu cattle are better adapted to the heat, most of the sexual activity seems to take place during the cooler hours of the night [89,95].

The presence of the male is another factor that affects the sexual behavior of the Zebu cow. The bull must be dominant enough to prevent cow–cow riding as well as younger bulls mounting. When a bull is used in breeding cows with synchronized estrus, the sexual behavior is grouped in a shorter period and the proportion of estruses with a shorter duration increases than in groups without a bull [95]. In addition, the male affects the start time of estrus [103].

## 4. Conclusions

Parturition is a very important moment, a determinant in the young´s life through the establishment of the cow–calf bond, which allows food and protection from the mother. In addition, the reproduction performance of the cow is also impacted at this time, as the period between parturition and the restart of ovarian activity is affected by the presence of the calf, the suckling events and the reserves of the cow, among others. Both the life of the calves and the reproductive activity of the cows are factors that can mean considerable economic losses for the producer. The knowledge of how the behavior and physiology of the animals affect these aspects is very important, without neglecting the aspects of wellbeing, both in the mother and in the offspring.

## Data Availability

Not applicable.
